# Screening for Latent Polycythemia Vera in Renal Cell Carcinoma–Associated Erythrocytosis

**DOI:** 10.15586/jkcvhl.v10i1.276

**Published:** 2023-04-22

**Authors:** Stephen E. Langabeer

**Affiliations:** Cancer Molecular Diagnostics, St. James’s Hospital, Dublin, Ireland

## Dear Editor,

Secondary erythrocytosis is a noteworthy, recurrent feature in patients with renal cell carcinoma (RCC) (1). Unlike secondary erythrocytosis, the most common form of primary erythrocytosis is the myeloproliferative neoplasm polycythemia vera (PV) characterized by the presence of the *JAK2* V617F somatic mutation in approximately 97% of cases and typically a low serum erythropoietin. Despite the requirement for excluding secondary causes in the diagnostic workup of PV (2), RCC patients with an erythrocytosis are sporadically referred for molecular detection of the *JAK2* V617F. An audit was therefore performed to determine the clinical and laboratory impact of *JAK2* V617F testing in patients with RCC-associated erythrocytosis.

At a center for hematological malignancy molecular diagnostics that receives greater than 2000 *JAK2* V617F diagnostic tests per annum, 24 requests for *JAK2* V617F mutation status were identified with clinical details provided of RCC (disease stage unknown) and either erythrocytosis and/or raised hemoglobin and/or raised hematocrit from January 2006 to December 2022 inclusive. The median and range of the hemoglobin and hematocrit were unknown as was whether the patients were receiving anti-vascular therapy. The median age of patients was 64 years (range 39–82 years) of which 15 patients (62.5%) were male. The *JAK2* V617F mutation was detected by an allele-specific PCR technique unchanged throughout the audit period with a sensitivity of detecting 5% mutant alleles (3). The *JAK2* V617F mutation was not detected in any of the 24 cases analyzed.

While RCC is a recognized cause of erythrocytosis (4), there exists a possibility of RCC-associated erythrocytosis and concomitant PV: a literature review identified only a single case of concomitant RCC and myeloproliferative neoplasm identified as *JAK2* V617F-positive essential thrombocythemia with an elevated platelet count, as opposed to an erythrocytosis (5). A previous study using denaturing gradient gel electrophoresis coupled with Sanger sequencing failed to identify any mutations of the *JAK2* JH2 domain (exons 10–16) (6). Despite molecular investigation having an inconsiderable impact on laboratory workload, this audit indicates that routine testing for latent *JAK2* V617F-positive PV in patients with RCC-associated erythrocytosis is unnecessary.
